# Novel insights into the molecular mechanisms underlying risk of colorectal cancer from smoking and red/processed meat carcinogens by modeling exposure in normal colon organoids

**DOI:** 10.18632/oncotarget.28058

**Published:** 2021-09-14

**Authors:** Matthew Devall, Christopher H. Dampier, Stephen Eaton, Mourad W. Ali, Virginia Díez-Obrero, Ferran Moratalla-Navarro, Jennifer Bryant, Lucas T. Jennelle, Victor Moreno, Steven M. Powell, Ulrike Peters, Graham Casey

**Affiliations:** ^1^Center for Public Health Genomics, University of Virginia, Charlottesville, VA, USA; ^2^Department of Public Health Sciences, University of Virginia, Charlottesville, VA, USA; ^3^Oncology Data Analytics Program, Catalan Institute of Oncology (ICO), L’Hospitalet de Llobregat, Barcelona, Spain; ^4^Colorectal Cancer Group, ONCOBELL Program, Bellvitge Biomedical Research Institute (IDIBELL), L’Hospitalet de Llobregat, Barcelona, Spain; ^5^Consortium for Biomedical Research in Epidemiology and Public Health (CIBERESP), Madrid, Spain; ^6^Department of Clinical Sciences, Faculty of Medicine, University of Barcelona, Barcelona, Spain; ^7^Digestive Health Center, University of Virginia, Charlottesville, VA, USA; ^8^Public Health Sciences Division, Fred Hutchinson Cancer Center Research Institute, Seattle, WA, USA

**Keywords:** colon organoids, microsatellite instability, smoking, single-cell deconvolution, weighted gene co-expression network analysis

## Abstract

Tobacco smoke and red/processed meats are well-known risk factors for colorectal cancer (CRC). Most research has focused on studies of normal colon biopsies in epidemiologic studies or treatment of CRC cell lines *in vitro*. These studies are often constrained by challenges with accuracy of self-report data or, in the case of CRC cell lines, small sample sizes and lack of relationship to normal tissue at risk. In an attempt to address some of these limitations, we performed a 24-hour treatment of a representative carcinogens cocktail in 37 independent organoid lines derived from normal colon biopsies. Machine learning algorithms were applied to bulk RNA-sequencing and revealed cellular composition changes in colon organoids. We identified 738 differentially expressed genes in response to carcinogens exposure. Network analysis identified significantly different modules of co-expression, that included genes related to MSI-H tumor biology, and genes previously implicated in CRC through genome-wide association studies. Our study helps to better define the molecular effects of representative carcinogens from smoking and red/processed meat in normal colon epithelial cells and in the etiology of the MSI-H subtype of CRC, and suggests an overlap between molecular mechanisms involved in inherited and environmental CRC risk.

## INTRODUCTION

Carcinogens in tobacco smoke and red/processed meat are known risk factors for colorectal cancer (CRC) and, tobacco smoke has been associated with tumors characterized by high microsatellite instability (MSI-H) [1, 2]. However, the molecular mechanisms underlying the relationship between these carcinogens and CRC are poorly understood. Elucidating these molecular mechanisms represents an important public health challenge.

Tobacco smoke and red/processed meat contain many known and potential carcinogens. Three important classes of carcinogens are commonly found: heterocyclic aromatic amines (HCA)s, polycyclic aromatic hydrocarbons (PCH)s, and nitrosamines. The genotoxic effects of the HCAs 2-amino-3, 8-dimethylimidazo [4, 5-*f*] quinoxaline (MeIQx) and 2-amino-1-methyl-6-phenylimidazo [4, 5-*b*] pyridine (PhIP), the PCH benzo(a)pyrene (*BaP*), and the nitrosamine *N*-nitrosodiethylamine (NDEA) have been studied in a variety of model systems [3, 4]. These carcinogens may reach the colonic mucosa either through the lumen of the gastrointestinal tract or through the circulatory system. Studies in CRC cell lines [5–8] have demonstrated important relationships between MelQx, PhIP, BaP, NDEA and oncogenic pathways, but the impact of these carcinogens on normal colon epithelial cells is not known.

Transcriptomic profiling of human colon biopsies has previously revealed gene expression differences associated with smoking [9] and red/processed meat [10]; however, these studies rely on the accuracy of subject reporting or were performed in patients who had already developed colon cancer. Furthermore, colon biopsies contain extensive cellular heterogeneity that may mask gene expression changes occurring in the cells of the stem cell niche of the colon crypt, where neoplastic changes are expected to originate [11]. Three-dimensional (3D) normal human colon organoids are an important model for the study of the stem cell niche of the colon crypt [12], benefitting from an increased cellular heterogeneity relative to CRC cell lines [12, 13] and the ability to control dose administration relative to most data collected from biopsies. We have previously shown that exposure of colon organoids to ethanol [14] or aspirin [15] can reveal candidate genes implicated in CRC risk through the analysis of bulk RNA-sequencing (RNA-seq) followed by single cell deconvolution to adjust for cellular content.

We leveraged RNA-seq and machine learning algorithms to elucidate the early transcriptomic and cellular effects of these carcinogens on normal human colonic epithelial cells. We identified differences in gene expression following a 24hr exposure of colon organoids to a single dosing of a carcinogens cocktail that included MelQx, PhIP, BaP, and NDEA. We observed a robust transcriptomic response in carcinogens exposed colon organoids that revealed selective changes in cell composition. We replicated a number of these differences in transcription in normal mucosal biopsies derived from current and never smokers using the University of Barcelona and University of Virginia RNA sequencing (BarcUVa-Seq) cohort [16]. Finally, we performed the first weighted gene co-expression network analysis (WGCNA) of colon organoids treated with carcinogens. We identified significant modules related to drug treatment, MSI-H tumor biology as well as modules driven by genes mapping to known CRC genome-wide association studies (GWAS) risk loci. Our results therefore extend the current understanding of how these carcinogens may impact normal colon crypt epithelial cell biology, and impact not only CRC etiology, but more specifically, the MSI-H subtype of CRC.

## RESULTS

### Carcinogen treatment of colon organoids leads to consistent patterns of differential expression

A large colon organoid biorepository was generated from colon crypts of healthy individuals (Supplementary Table 1). RNA-seq was generated on 37 independent, subject-derived organoid lines treated with carcinogens or vehicle control (see Supplementary File 1 for quality control metrics). We performed hierarchical clustering on our dataset, where we found that all sample pairs fell within two large branches, except for one, which was subsequently removed from downstream analysis (Supplementary Figure 1). All subsequent analyses were performed on the remaining 36 organoid lines.

Note that previous studies involving colon organoids are often associated with much smaller sample sizes, typically ten or less [13, 17–20]. We performed differential expression analysis on pseudo-cohorts of multiples of five pairs generated by random sampling. We found that at five pairs, the lowest number of DEGs identified (24) was ~14.8-fold less than the maximum number of DEGs identified within that subset (Supplementary Table 2). This suggests that most published studies involving organoids may be too small to provide robust data.

In our dataset (*n* = 36) a mixed-effects regression [21] revealed that 2,649 DEGs were associated with carcinogen treatment, and identified expected findings for genes such as , cytochrome P450 family 1 subfamily A member 1 (*CYP1A1*; (*P*_Bonferroni_ = 3.75E^−14^)) and cytochrome P450 family 1 subfamily B member 1 (*CYP1B1*; (*P*_Bonferroni_ = 1.71E^−17^)) [22]. We observed no impact of colon location (right versus left colon) following stratified analysis (data not shown) in contrast to our previous study of ethanol exposure in colon organoids [14]. We technically validated a subset of these genes (*n* = 5/5) using qPCR in a subset (*n* = 4) of samples (Supplementary Table 3).

### Carcinogen exposure of normal colon organoids leads to cellular composition changes

**Figure 1:**
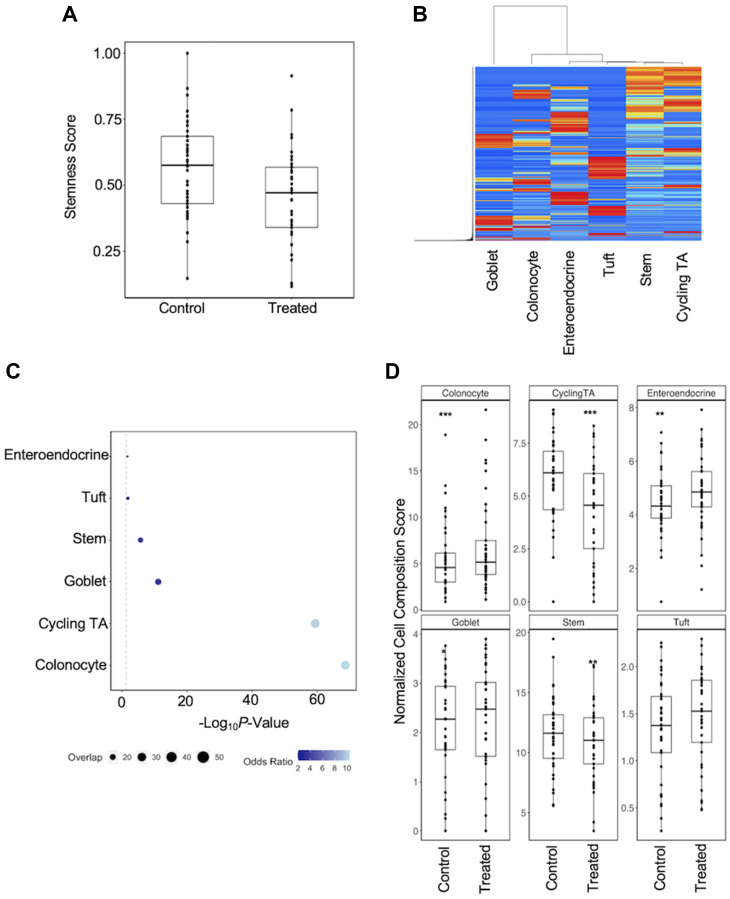
Regression analysis of cell composition differences in response to carcinogen exposure. (**A**) Carcinogens exposed organoids were associated with a reduced stemness index. (**B**) Heatmap of gene expression for signature matrix genes shows that selected genes are able to stratify cell types in single-cell data. (**C**) Enrichment analysis to determine overlap between DEGs identified by regression on cell score in colon organoids and known markers of cell types. Larger circles indicate a greater overlap between total number of marker genes and those identified as being significant in each regression. Increasing odds ratios generated from enrichment Fisher’s Exact tests is represented as a transition from light to dark blue. (**D**) Cell score regression analysis between treatment conditions: ^*^ *P* < 0.05; ^**^ *P* < 0.01; ^***^ *P* < 0.001.

To estimate the effect of carcinogens on cell composition, we compared stemness scores in each organoid pair between conditions [23]. Analysis of these scores has previously shown that stemness indices in primary tumors are greater than those of normal tissue adjacent to the tumor, including in colon and rectal cohorts [24]. Surprisingly however, treatment with carcinogens led to an overall reduction of stemness in colon organoids (*P* = 6.13E^−14^; 36 pairs). This was consistent across all 36 carcinogen-treated organoid pairs (Figure 1A). To confirm the apparent relative increase in differentiated cells, we downloaded and processed scRNA-seq data derived from colon biopsies of healthy individuals [25] and used a machine learning approach to infer cell type composition in our dataset (Figure 1B) [26]. We previously applied a similar approach to study the effect of short term ethanol exposure on cellular composition in colon organoids [14]. We generated cell proportions for six epithelial cell types. Of note, the signature matrix generated here contained 57.3% of the gene expression markers used for the generation of a high-throughput method for the assessment of cell composition recently developed for intestinal organoids [27]. We performed regression on cell score for each cell type and found that single-cell expression markers from Smillie et al., [25] were significantly overexpressed and enriched in our dataset (Figure 1C). Following this, we performed a mixed-effects regression analysis of cell score between treatment conditions (Figure 1D). We identified a significant reduction in transit amplifying (TA) (*P* = 2.98E^−07^; 28 pairs) and stem cells (*P* = 1.98E^−03^; 24 pairs), and a significant increase in colonocytes (*P* = 1.69E^−05^; 30 pairs), enteroendocrine (*P* = 1.29E^−03^; 24 pairs) and goblet cells (*P* = 0.016; 19 pairs). However, we were only able to replicate one of these findings following deconvolution of the BarcUVA-seq dataset after performing a regression for cell composition on smoking status. Analysis of this dataset revealed a significant increase in goblet cells (*P* = 0.039) in the colon of smokers (*n* = 60) versus non-smokers (*n* = 223), but not other cell composition differences that were identified in organoids.

### Extensive gene expression differences in response to carcinogen exposure following cell type adjustment

**Figure 2:**
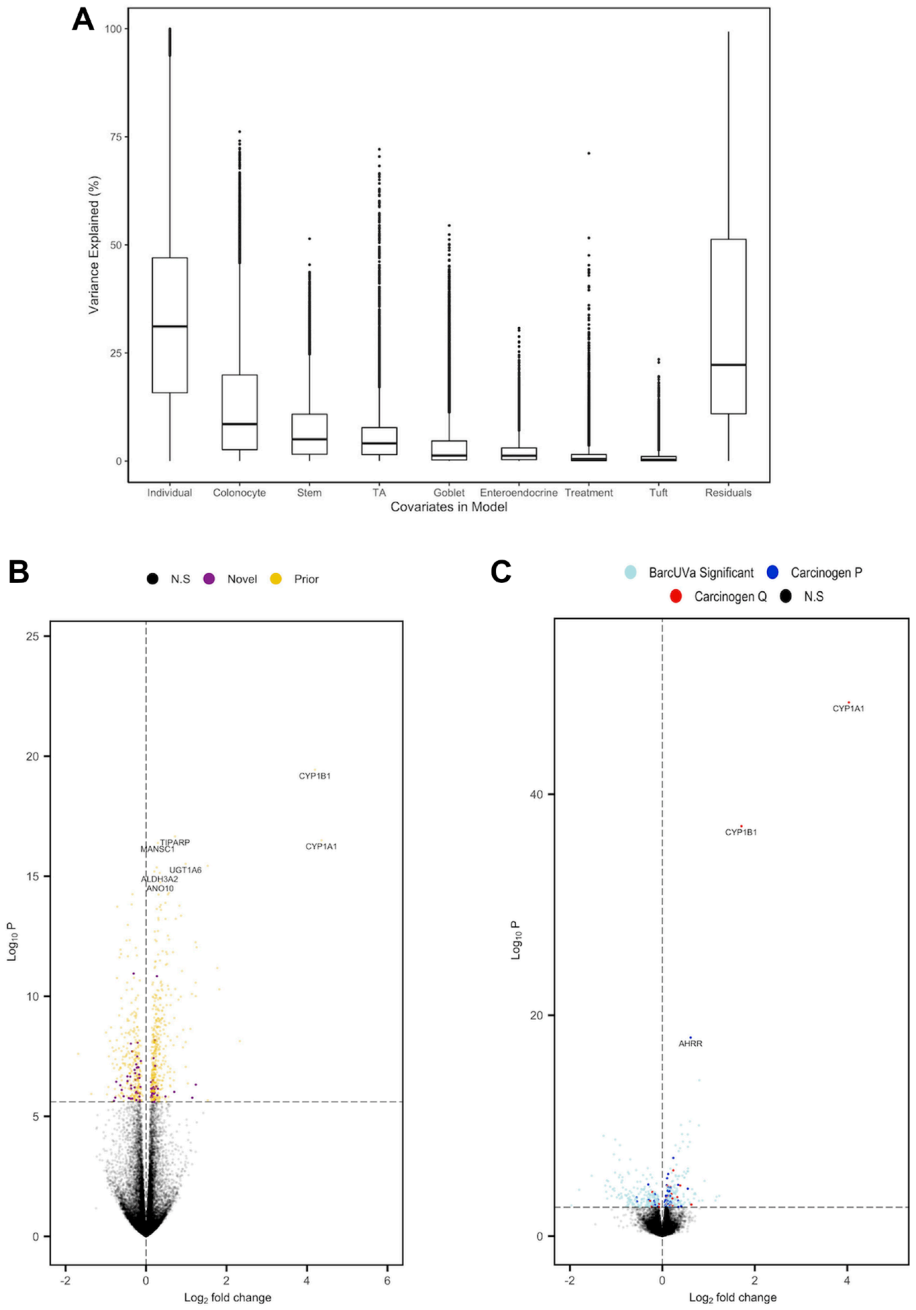
Summary of analysis of carcinogen exposure of organoids following adjustment for cell composition. (**A**) Boxplot to show the proportion of gene-wise variance explained by each covariate within the mixed-effects regression model. (**B**) Volcano plot of carcinogen DEGs. ‘Prior’ and ‘Novel’ denote genes that were and were not previously identified in original analysis respectively (**C**) Volcano plot of BarcUVa-Seq analysis. DEGs only identified in BarcUVa-Seq are denoted light blue, while genes that were also present nominally (dark blue) and following Bonferroni correction (red) in carcinogen analysis are also shown. N.S denotes genes that were not significant.

We have previously shown that adjusting for cell composition enriches for biological signal and reduces the reporting of DEGs driven by cellular heterogeneity [28]. Thus, we incorporated cell scores into our regression model and measured the extent of variation in gene expression that could be attributed to cell composition (Figure 2A). We found a number of genes whose variance was mostly explained by carcinogen treatment, such as *CYP1B1* (71.17%) and steroid 5 alpha-reductase 1 (*SRD5A1*; (51.61%)), while variation attributed to cell markers were accurately reflected by changes in cell composition for that cell type. For example, 70.42%, 66.28% and 64.22% of the variation in TA cell markers baculoviral IAP repeat containing 5 (*BIRC5*), centrosomal protein 55 (*CEP55*) and centrosomal protein I (*CENPI*) [25] could be explained by changes in TA cell composition. We performed a mixed-effects regression across all samples by modelling sample pair and cell scores as the random and fixed effects respectively, which led to the identification of 738 DEGs following Bonferroni correction (Supplementary File 2). Most (663/738) were also present prior to deconvolution, while 75 were only significant after adjustment for cell composition (Supplementary File 2; Figure 2B). Of these 738, 200 had not been previously reported as being affected by any of the carcinogens in our cocktail when compared to those collated in the Comparative Toxicogenomics Database [22]; the most significant of which was an increase in anomactin 10 (*ANO10*; (P_Bonferroni_ = 3.59E^−11^)), a gene which has been found to affect calcium signaling in mouse intestinal epithelial cells [29].

### Replication of findings in CRC risk factors comprised of carcinogens

To contextualize the carcinogens-related DEGs we identified, we analyzed RNA-seq data from a large population-based cohort of normal colon mucosal biopsies (BarcUVa-Seq) cohort (Supplementary File 3). We have previously performed deconvolution analyses of this dataset [15] and used cell scores those cell scores to adjust for cell composition within our subsequent regression model. Given that our interest only lay within the 738 DEGs identified in colon organoids, we set a validation threshold at *P* < 0.05 for these genes. To the best of our knowledge, the BarcUVa-Seq cohort represents the largest dataset reported for smoking and red/processed meat consumption in normal colon biopsies taken at colonoscopy (Supplemental File 3). Following adjustment for cell composition we were able to replicate 87 of 738 carcinogen DEGs at this validation threshold (Supplementary File 2; Figure 2C), of which 27 also passed multiple testing corrections (FDR = 0.1). One-way Fisher’s exact test determined that the extent of overlap for significant genes was greater than expected by chance (*P* = 4.86E^−03^). Of note, we were only able to replicate 30 of our findings in a separate, combined analysis of red/processed meat consumption in the BarcUVa-seq cohort (Supplementary File 2).

### Overlap between DEGs following carcinogens exposure and genes mapping to CRC GWAS loci

To determine if there was any potential relationship between molecular mechanisms underlying inherited and environmental risk for CRC, we intersected DEGs identified following carcinogens exposure with genes mapping to CRC GWAS loci. We downloaded index SNPs from the GWAS catalogue [30] and found that of the 738 carcinogen-related DEGs, 61 genes mapped within 1Mb of the index SNP of 37 CRC GWAS loci (Odds Ratio = 1.27, *P* = 0.049) (Supplementary Table 4).

### WGCNA reveals altered patterns of co-expression following carcinogen treatment

Genes rarely act in isolation, and expression of genes within related pathways are usually coordinated in such a way that they can be identified using systems level approaches. We have previously found that WGCNA led to the identification of modules driven both by aspirin-related genes and CRC loci in a similarly sized cohort of aspirin treated colon organoids [15]. Here, we generated a network of gene co-expression in our colon organoid model using WGCNA [31] and determined whether modules comprised within this network were differentially associated with carcinogens exposure (Supplementary Figure 2).

In total, we identified 55 modules of co-expression. Of these, seven modules were considered for further analysis following additional quality control measures (see Methods). These seven modules contained a total of 28.32% of the 738 DEGs identified in our single-gene approach, despite comprising only 8.65% of all genes in the network. Module functionality was determined through enrichment analysis of Gene Ontology biological processes (Supplementary File 4). For each significant module, a node profile was generated by determining representative hub genes (Table 1). Gene hubs were defined by a “fuzzy” measure of module membership. The greater the module membership, the greater the connectivity of the gene within the module.

**Table 1:** Summary of significant modules identified in WGCNA that passed quality control tests and were enriched for protein-protein interactions

Module	*t*-value	*P*_Bonferroni_	Gene Significance and Module Membership	No. CRC GWAS Genes	PPI	Hub Genes
lightsteelblue	31.949	9.62E-26	0.360 (*P* = 1.50E-08)^+^	15 (233)^*^	1.00E-16	*PIP4K2C, HGD, TMEM127, ITSN2, SUSD6, NUDT16, CCRL2, CDX2, MUC13, CYP1A1*
bisque4	–24.523	6.84E-22	0.430 (*P* = 1.80E-06)	11 (114)	0.046	*THOC, TKFC, TMEM234, FBL, EXOSC7, ZNF318, SMARCD2, COROA1, MAPK15, KISS1R*
coral1	–16.215	4.14E-16	0.150 (*P* = 1.80E-03)	37 (432)	1.00E-16	*RPL35A, RPL32, RPS18, NACA, RPL5, RPL11, RPL7A, RPL6, RPL39, ECI2*
skyblue4	–15.882	7.86E-16	0.420 (*P* = 1.30E-03)	7 (56)	1.00E-16	*FDPS, MVK, ACAT2, LSS, DHCR7, NSDHL, FASN, ERG28, ETHE1, FDFT1*
coral	–14.934	5.18E-15	0.110 (*P* = 0.020)	39 (449)	1.00E-16	*TCOF1, PFAS, MCM3, MCM7, GEMIN4, MCM4, MCM6, PRMT1, POLD2, RRP1*
darkolivegreen	–8.313	4.64E-08	0.220 (*P* = 9.30E-03)	14 (139)	6.29E-10	*TNK2, PPP1R13L, RASSF7, PLEKHH3, AGPAT2, UBE2C, LTBP4, TP53I11, PAK4, BLVRB*
plum4	5.249	4.15E-04	0.120 (*P* = 0.029)	21 (330)	1.00E-16	*ORC5, EIF3J, FBXO45, ABHD13, ZMPSTE24, CYCS, LEPROTL1, LARP4, KPNA3, BAG5*

^+^Significance of correlation between gene significance and module membership for genes within a module. ^*^Total number of genes within a module.

Lightsteelblue was the most significant module associated with carcinogen treatment (*P*_Bonferroni_ = 9.62E^−26^), containing fourteen of the top twenty most significant DEGs. Further, 40 of the 233 genes comprising lightsteelblue were associated with smoking in BarcUVa-Seq (*P* = 0.05), including *CYP1B1*, TCDD inducible poly(ADP-Ribose) polymerase (*TIPARP*) and *CYP1A1*. Genes within this module were generally overexpressed following carcinogen treatment. We found that the most representative hub gene within lightsteelblue was phosphatidylinositol-5-phosphate 4-kinase gamma (*PIP4K2C*), which was in the vicinity of 12q13.3/rs4759277, a known CRC GWAS region [32]. Similarly, we found that La ribonucleoprotein 4 (*LARP4*), one of the ten hub genes identified in the plum4, was also in the vicinity of 12q13.3/rs4759277 [32–34]. Pathway enrichment analysis revealed that changes in the module’s eigengene may have effects on various metabolic processes as well as others such as posttranscriptional regulation of gene expression (FDR = 2.00E^−03^), regulation of translation (FDR = 3.5E^−03^) and regulation of cell cycle (FDR = 7.80E^−03^).

Of the other four modules considered, coral was determined to be of most interest, due to its potential biological relevance. Hub gene analysis of this module revealed a relevant, highly connected node at DNA polymerase delta 2, accessory subunit (*POLD2*). DNA polymerase epsilon, catalytic subunit (*POLE*) and DNA polymerase delta 1, catalytic subunit (*POLD1*) were also present within this module; germline and somatic mutations of these genes have been associated with CRC [35]. This module was negatively associated with carcinogen treatment (*P*_Bonferroni_ = 5.18E^−15^) and enriched for MSI-H related pathways such as cellular response to DNA damage stimulus (FDR = 1.47E^−18^), DNA repair (FDR = 3.56E^−15^) and MMR (FDR = 8.42E^−05^). Coral contained numerous genes previously associated with MSI-H status. Many of these genes were only nominally associated with carcinogen exposure in our single gene analysis such as mutL homolog 1(*MLH1*), mutS homolog 2 (*MSH2*), mutS homolog 6 (*MSH6*), and replication factor C subunit 3 (*RFC3*), highlighting the importance of WGCNA in helping to elucidate underlying biological mechanisms.

## DISCUSSION

To our knowledge this is the first to study the impact of carcinogens from smoking and red/processed meats on normal epithelial cells of the colon crypt stem cell niche, the expected target cell population for the origin of neoplastic changes [11]. Previous studies aimed to determine the transcriptomic response of smoking/red meat in the colon using patients already presenting with CRC [9, 10], while studies of the individual chemical constituents of our carcinogen cocktail have been primarily performed in CRC cell lines [5–7, 36, 37]. These are unlikely to reflect the normal response of colon crypt epithelial cells to environmental factors. This, coupled with the large increase in power present within our study compared to other *in vitro* analysis of carcinogens may, in part, explain why 200 of the 738 DEGs identified in our dataset were deemed to be novel [22].

The use of single-cell deconvolution to estimate cell composition has become increasingly popular in recent years [26]. Recently, this has even led to the development of high-throughput methods based on targeted RNA-seq primarily for the evaluation of cell composition in intestinal organoids [27]. Here, we used a well-established method for the estimation of cell composition [26], the signature matrix of which displayed considerable overlap with targeted approaches for intestinal organoids [27]. Our study was performed in a sample set of 36 patient-derived normal colon organoids where we also observed significant differences in the abundance of stem, TA, goblet, colonocyte and enteroendocrine cell populations. Previous studies have reported increased goblet cell numbers in other epithelial tissues of smokers [38], and we were able to replicate the goblet cell finding in BarcUVa-Seq mucosal biopsies of smokers in BarcUVa-Seq mucosal biopsies of smokers. That we were unable to replicate other cell composition changes in the BarcUVa-Seq cohort may be due to challenges associated with self-report data, and limited *in vitro* exposure conditions versus lifetime exposure. These limitations may also partly explain why stronger replication of DEGs were not seen in the BarcUVa-Seq cohort. Further independent studies are warranted to determine the effect of these carcinogens on cellular composition within the colon crypt.

Increased expression of *CYP1A1* and *CYP1B1* were among the most significant transcriptomic differences identified in colon organoids in response to carcinogens exposure. These findings are consistent with published reports and underscore the role of these two genes in the cellular response to HCAs and PCHs [3]. Altered expression of these genes has previously been associated with smoking [3], and both genes also showed increased expression in relation to smoking use in our BarcUVa-Seq mucosal biopsy study. However, not all findings were consistent with the literature. For example, increased expression of aryl-hydrocarbon receptor repressor (*AHRR*) among smokers is one of the most consistently reported in the literature [3] and was significantly overexpressed in BarcUVa-Seq subjects who smoked, however, it was only nominally increased in colon organoids exposed to carcinogens. Importantly *AHRR* is highly expressed in T cells of the colon [39], but only expressed at low levels in colon organoids which consist only of epithelial cells, which may help to explain this inconsistency. Our studies highlight the impact of carcinogens on epithelial cells of the stem cell niche of colon crypts directly. Advances in organoid co-culturing methods, may help clarify effects on other cells of the colon.

Our study highlights a potential mechanistic overlap between genes implicated in inherited and environmental risk of CRC. We observed a large number of DEGs responsive to these carcinogens with genes mapping within CRC GWAS loci. Of note, we also observed an overlap between genes responsive to ethanol/alcohol exposure in organoids and genes mapping within GWAS loci [14]. CRC GWAS have led to the identification of >140 genomic loci [32]; however, relevant target genes have been identified for only a few of these loci [40–42]. This is in part due to the fact that the vast majority of GWAS variants are believed to influence disease through modulation of enhancer activity, subsequently impacting gene expression. As these variants rarely fall within coding regions, interpretation of the gene target has often proven challenging. Our comparison of genes identified through both our single-gene approach and WGCNA with genes that mapped to CRC GWAS loci revealed a considerable overlap implying a potential relationship between molecular mechanisms underlying inherited and environmental risk for CRC. In our single-gene analysis, we identified 61 genes from 37 GWAS loci that overlapped carcinogens-related DEGs. Network analyses have been used in research into other complex genetic traits to identify candidate genes involved in GWAS [43]. We have also previously made use of WGCNA to unravel the relationship between aspirin and CRC risk loci in colon organoids [15]. From our WGCNA, we identified candidate GWAS-related genes *LARP4* and *PIP4K2C* as respective hub genes for the plum4 and lightsteelblue modules. In other model systems, *LARP4* has been implicated in mRNA stabilization [44]. Consistent with this, the plum4 module was enriched for numerous regulatory processes including posttranscriptional regulation of gene expression. Elucidating how *LARP4* may increase mRNA stability within the colon, as well as which targets may be most affected *LARP4* overexpression may provide further insight into its potential role in CRC, particularly given the important role for mRNA stabilization in the shaping of the cancer transcriptome [45]. With regards to *PIP4K2C*, our single-gene analysis also reveals that it was significantly increased in carcinogen exposed organoids. A strong link between *PIP4K2C* and CRC has yet to be defined; however, inhibition of *PIP4K2C* has been shown to increase immune system activation, making it a potential target for cancer immunotherapy [46]. WGCNA also led to the identification of a highly connected node at *POLD2* in the coral module. *POLE* and *POLD1* were also present within this module, and both inherited variants and somatic mutations of these genes have been associated with CRC [35]. Further studies are warranted to determine whether the comparative approaches we describe can be used to interrogate the function of GWAS risk variants and accelerate discovery of GWAS-related biology.

The coral module was enriched for a number of genes associated with MMR. Increasing evidence has demonstrated a strong association between smoking and MSI-H CRC tumor development [1, 2]. MSI-H tumors are driven by reduced expression of specific DNA MMR genes [28], and the average expression of genes within the coral module were reduced in colon organoids exposed to carcinogens. The coral module also contains *MLH1*, *MSH2* and *MSH6*, inherited pathogenic variants for which are well known to be causal in Lynch syndrome. Importantly, somatic hypermethylation and downregulation of *MLH1* is associated with the majority of MSI-H tumors. We recognize that while these findings are of interest, we do not know if the observed effects on gene expression by these carcinogens are maintained over longer time periods, or are causal for MSI-H CRC tumors.

These results may also have important implications for red meat and processed meat consumption and CRC risk [47, 48]. While some studies have supported an association between red meat consumption and increased CRC risk [47, 49], this result is not consistent [50, 51]. The relationship between red/processed meats and CRC subtypes also remains controversial with some studies suggesting a positive relationship between red meat consumption and MSI-H tumors (12), while others do not [52, 53]. If confirmed, our study may provide some insight into the molecular mechanisms underlying the relationship between carcinogens present in tobacco smoke and red/processed meat and MSI-H CRC tumors. However, here we performed an analysis of red/processed meat and found somewhat limited overlap with carcinogen DEGs. This may be due to a number of reasons. For example, while dietary questionnaires are a powerful tool, accurate reporting is often challenging as dietary habits change over time and in some cases, are based on recall.

Our study is not without limitations. With regards to the experimental design: a single time point/dose was used to model the effect of carcinogens. In this way, our study does not model the infrequent dosing of carcinogens likely observed through smoking or dietary intake. The selection of dose for each compound was similar to doses chosen across numerous previous studies in different cell lines [5, 7, 36, 37, 54–57]. However, we note that the carcinogens doses chosen for this study are likely orders of magnitude greater than would be expected to be found in the colon from tobacco smoke inhalation and/or daily consumption of red/processed meats. Future studies would greatly be improved by conducting pharmacodynamic experiments to determine the concentration of these compounds that enter the large intestine [6, 58]. A single-dose approach was chosen to allow for more sophisticated analysis and improved confidence in reporting of results owing to an increased power to detect differential expression. We highlight the importance of such a study design through permutation analysis, which shows that DEG reporting in common sized organoid designs are highly variable. We note that this may create a constraint on the broad applicability of our analysis. Further, the use of a cocktail of carcinogens may lead to potential synergism and/or negation of the effects of individual genes or pathways associated with each component. It is worth noting that these combinatorial effects would also occur in the setting of smoking and diet. In addition, we assume here that metabolism of each component within the carcinogen cocktail occurs similarly to that occurring within other cell lines with high *CYP1A1* and *CYP1B1* expression [59]; however metabolomic screening was not performed on these colon organoids. Changes in compound metabolism may affect the overall gene expression response, and such considerations should be made in future studies. Finally, with regards to the choice of validation: our use of BarcUVA-seq to replicate our findings is not ideal. BarcUVA-seq is the largest RNA-seq colon biopsy cohort with smoking and dietary information available, and we were able to use it to replicate a number of the observed differences in our organoid treatment. However, only a limited number of DEGs were replicated in our analysis. This could be driven by limitations discussed earlier. However, despite these limitations, we did observe an enrichment for DEGs between colon organoids exposed to these carcinogens and those seen in smokers of a large colon biopsy dataset.

In conclusion, we identified extensive gene expression and cellular composition differences following exposure of normal colon organoids to carcinogens commonly found in tobacco smoke and/or red/processed meat. We provide data suggesting an overlap between genes implicated in inherited and environmental CRC risk, that may help accelerate discovery of biological mechanisms underlying risk. Through WGCNA, we also identified a potential molecular mechanism underlying the relationship between these carcinogens and MSI-H CRC etiology. These discoveries provide novel insights into CRC etiology and reveal several avenues for future research.

## MATERIALS AND METHODS

### Subject recruitment and exclusion criteria

Subjects scheduled for screening or surveillance colonoscopies who agreed to voluntary participation in this study were enrolled after providing informed consent under an approved Institutional Review Board protocol at the University of Virginia (IRB-HSR #19439 and IRB-HSR #15274). Subjects were recruited between July 2017 and March 2019 and agreed to donate biopsies from both right and left colon. Subjects were excluded from this study if they had a personal or family history of CRC, a personal history of inflammatory bowel disease, or high-risk polyps at the time of colonoscopy. Most (26 of 37) subjects had no polyps at the time of colonoscopy, and the remainder had three or fewer tubular adenomas each less than 10 mm in largest dimension. All procedures were performed in accordance with relevant guidelines and regulations and were consistent with those required by both the National Institutes of Health and the University of Virginia.

### Epidemiologic data collection for BarcUVa-Seq

BarcUVa-Seq data was processed as in the original study [16]. For the purpose of this study, additional epidemiologic data were collected. To collect information on red and processed meat, a self-administered food frequency questionnaire was adapted from one previously validated [60]. This questionnaire was used to asses dietary intake at the time of subject recruitment. The questionnaire collected information on the consumption of multiple dietary variables from the preceding year. For the purpose of this study, red meat (grams/day) was taken as the sum of duck, veal, ox, cow, beef, pork and lamb. Processed meat (grams/day) was taken as the sum of sausages, hamburgers, hot dogs, pâté, liver and the percentage of meat present in mixed dishes. For smoking: “never smokers” were defined as those who have smoked less than 100 cigarettes or 360g tobacco within their lifetime; “current smokers” answered yes to a question regarding whether they currently smoked either now, or within the past month; “former smokers” had exceeded the limits for “never smokers”, were not defined as “current smokers” and had also smoked at least one cigarette regularly for a period of at least six months.

### Establishment and passaging of colon organoids

Normal 3D colon organoids included in this study were developed from biopsies of either right or left colon using a modification of the method described by Sato, et al. [12]. Biopsies were obtained immediately distal to the hepatic flexure (right colon) or immediately distal to the splenic flexure (left colon). Whole crypts were isolated by gentle mechanical disruption and embedded in Matrigel [12]. Growth media included advanced DMEM/F12, 100 U/ml penicillin, 100 μg/ml streptomycin, 10 mM Hepes, 1x N2, 1x B27, 1x GlutaMAX, 1.25 mM N-acetylcysteine, 10 nM gastrin, 50% L-WRN conditioned media, 500 nM A83-01, 10 uM SB202190, 10 mM nicotinamide, 50ng/ml EGF, and 10 μM Y27632. Colon organoids were grown and passaged as needed in 48-well culture plates, as previously described [12–14].

### Exposure of colon organoids to carcinogens

Three days prior to the initial exposure, organoids were passaged. One set of wells for each organoid line was exposed to the carcinogen cocktail (5 μM MelQx, 5 μM PhIP, 1 μM BaP, and 10 mM NDEA), and a matching set of wells for each organoid line was exposed to a vehicle control (1.5 μL DMSO and 2.52 μL acetone per 10 mL growth media). Previous studies have found similar concentrations of PhIP in human colon after a single dosing of 70–84 μg [61], though this dose is much greater than the expected concentrations of PhIP in red/processed meat and smoking. Similar concentrations of MeIQx have previously been considered for pharmacodynamic studies of the intestine [54]. The dose for BaP was also within range of those previously considered [55–57]. Doses of PhIP, MeIQx and BaP chosen here are lower than the cytotoxic range previously indicated for these compounds in other cell lines [56, 62], while the dose of NDEA was below the range previously considered as genotoxic for investigations into CRC cell lines [7, 37]. However, we note that the doses chosen for this study are orders of magnitude higher than would be expected to be found in the colon from tobacco smoke inhalation and/or daily consumption of red/processed meats. The 24 hr time period was chosen because a longer period >48 hrs began to impact cell growth (data not shown). After 24 hours of growth, residual media was removed, Matrigel was mechanically disrupted, and 200 μL of RNA Lysis Solution RA1 (without TCEP) (Clontech/Machery-Nagel RNA XS Kit) was added to each well. The contents of each well were then collected in a sterile Eppendorf tube. Tubes were briefly vortexed and cell pellets stored at –80°C prior to RNA extraction.

### RNA extraction and sequencing

Total RNA was extracted using NucleoSpin RNA XS Kit. All samples used for library preparation had RNA integrity numbers above 9.8, as measured by Agilent 4200 Tapestation. Library preparation and RNA-seq was carried out according to Illumina protocols at the Northwest Genomics Center of the University of Washington. Paired-end, 100 bp sequencing was performed using the Illumina NovaSeq 6000. Reads were trimmed and aligned to GENCODE v29 reference genome using STAR [63]. On average 75% of reads uniquely mapped, yielding a median of 33.5 million reads per sample. Genes were quantified using HTSeq [64]. Data is available under accession number GSE174650.

### qPCR of colon organoids

RNA for qPCR was isolated as described above. RNA concentration was determined on a Qubit fluorometer (Thermo-Fisher). A minimum of 2000 ng of Total RNA was reverse transcribed to first-strand cDNA using the High-Capacity cDNA Reverse Transcription Kit (Thermo-Fisher). First-Strand cDNA was used for Taq-Man qPCR monitored on a QuantStudio Real-Time PCR analyzer (Thermo Fisher). Pre-Designed TaqMan Gene Expression Assays (Thermo Fisher) were used for quantification of several genes. Glucuronidase beta (*GUSB*) was used as a control gene to determine delta-CT values, which were then used as input for a paired empirical Bayes regression [65].

### Calculation of cell type composition scores

For colon organoids, raw unique molecular identifier count data from the epithelial cell subset of a single cell RNA-seq dataset of healthy colon biopsies was downloaded [25]. Count matrices were imported into Seurat V3 [66], and processed as previously described [14]. The dataset was down sampled to reduce computational burden. When available, mature cell populations were selected to increase the variation observed between cell populations. The cell identities defined by the original study authors were used, except that “Best4+enterocytes” and “enterocytes” were merged and labeled “colonocytes”. A total of 2,593 cells remained across six populations (colonocytes, cycling transit-amplifying (TA), enteroendocrine, goblet, stem and tuft cells). Transcripts per million were generated for each cell and uploaded into CIBERSORTx [26]. Analysis parameters are reported in Supplementary Table 5. BarcUVa-Seq data was deconvoluted for use as validation in a previous study [15], and the same cell scores were used here.

### Mapping genes to CRC GWAS loci

CRC GWAS index SNPs were downloaded from the GWAS catalog [30]. Genes with at least one nucleotide of one exon overlapping a 1 MB interval centered on the index SNP were included in the analysis. The genomic location of SNPs was based on their hg38 coordinates. BiomaRt [67, 68] was used to determine GrCH38 gene coordinates of nearby genes.

### Statistical analysis

All statistical analysis was carried out in R, version 4.03 [69]. A mixed-effects model was used for differential expression analysis [70, 71]. For identification of DEGs in the organoid model, a strict Bonferroni correction was set (*P*_Bonferroni_< 0.05). As Dream and variancePartition incorporate precision weights from limma/voom [65, 72], differential expression analysis for BarcUVa-Seq was performed using the voom method and an empirical Bayes regression on moderated t-statistics [65]. For replication of main findings in BarcUVa-Seq a validation threshold was set at (*P* < 0.05). Benjamini-Hochberg corrected *Q*-values were also generated based on the full regression model. The following regression models were used in the analysis of (1) colon organoids, (2) BarcUVa-Seq smoking (never versus current), (3) BarcUVa-Seq meat:

1. *Expr ~ Pair + Scores + Treatment*
2. *Expr ~ Sex + Scores + Age + Batch + Location + Treatment*
3. *Expr ~ Sex + Scores + Age + Batch + Location + Smoking + Meat*

where *Expr* = gene count, *Pair* = sample ID, *Scores* = cell composition, *Sex* = sex, *Batch* = sequencing batch, *Location* = colon location (right/left/transverse), *Treatment* = condition, Age = age at biopsy, *Smoking* = factor variable (current, former, never), *Meat* = 4^th^ versus 1^st^ quartile of the average of processed and red meat consumption.

Stemness scores were generated using an approach outlined previously [24]. Cell score and stemness regression analyses were performed using mixed-effect models in the lme4 package [73]. For cell composition analysis in BarcUVa-Seq, a linear regression was used with sex, batch, age and location as adjustment covariates.

For analysis of sample size considerations, sample pairs were randomly split into factors of five. A total of 20 permutations of sample pairs were considered for each set of five. Given the large differences in performance with regards to processing time, limma/voom was preferred to Dream.

### WGCNA of colon organoids

Prior to voom transformation, the colon organoid RNA-seq dataset was filtered to only include genes present in our single gene analysis (20,255). Genes were then converted to trimmed mean of M-values. Adjustment for cell score was carried out using the RemoveBatchEffect function in limma [65]. WGCNA was performed across all samples under default settings [31], with a few notable exceptions: bi-midweight correlation was used; the network was raised to a soft thresholding power of five; signed-hybrid parameters were specified throughout; module size was set to 20; a deep split of four was used and resulting modules with correlation greater than 0.8 were merged. Given the paired nature of the study design, significant differences in module eigengenes across treatment conditions were calculated using a linear mixed-effects model in lme4 [73]. Only modules where gene significance and module membership were significantly correlated were considered for further investigation. Given our paired design, gene significance was calculated by using the absolute value of the test-statistic generated in our Dream analysis. These adaptations to WGCNA have previously been defined [74]. Module gene lists were analyzed in STRING, where PPI networks were constructed [22]. Interactions for PPI were sourced across using all available evidence, under default settings. Modules that displayed significant enrichment for PPI and passed other quality control measures were considered for functional annotation by calculating enrichment of Gene Ontology terms [75] in STRING [22].

### Transcript profiling

Raw data generated for this manuscript has been uploaded to Gene Expression Omnibus and is available for download using accession number: GSE174650. Details for access for BarcUVa-Seq can be found in the original manuscript publication [16].

## SUPPLEMENTARY MATERIALS











## Abbreviations

3D: three-dimensional
AHRR: aryl-hydrocarbon receptor repressor
ANO10: anomactin 10
BarcUVa-Seq: University of Barcelona and University of Virginia RNA sequencing project
BaP: benzo(a)pyrene
BIRC5: baculoviral IAP repeat containing 5
CEP55: centrosomal protein 55
CENPI: centrosomal protein I
CRC: colorectal cancer
CYP1A1: cytochrome P450 family 1 subfamily A member 1
CYP1B1: cytochrome P450 family 1 subfamily B member 1
DEG: differentially expressed gene
GUSB: glucuronidase beta
GWAS: genome-wide association studies
HCA: heterocyclic aromatic amine
LARP4: La ribonucleoprotein 4
MeIQx: 2-amino-3, 8-dimethylimidazo[4, 5-*f*]quinoxaline
MLH1: mutL homolog 1
MMR: mismatch repair
MSH2: mutS homolog 2
MSH6: mutS homolog 6
MSI-H: microsatellite instability high
NDEA: *N*-nitrosodiethylamine
PCH: polycyclic aromatic hydrocarbon
PhIP: 2-amino-1-methyl-6-phenylimidazo [4, 5-*b*] pyridine
PIP4K2C: phosphatidylinositol-5-phosphate 4-kinase gamma
POLD1: DNA polymerase delta 1: catalytic subunit
POLD2: DNA polymerase delta 2: accessory subunit
POLE: DNA polymerase epsilon, catalytic subunit
PPI: protein-protein interaction
qPCR: Quantitative Real-Time PCR
RFC3: replication factor C subunit 3
RNA-seq: RNA-sequencing
SRD5A1: steroid 5 alpha-reductase 1
TA: transit-amplifying
TIPARP: TCDD inducible poly(ADP-Ribose) polymerase
WGCNA: weighted gene co-expression network analysis
