# Supplementary material for: Novel insights into the molecular mechanisms underlying risk of colorectal cancer from smoking and red/processed meat carcinogens by modeling exposure in normal colon organoids
Source: Oncotarget. 2021 Sep 14;12(19):1863–77. doi: 10.18632/oncotarget.28058 (PMC8448508; doi:10.18632/oncotarget.28058)
Supplement: Supplementary file 1 [file oncotarget-12-1863-s001.pdf]

Novel insights into the molecular mechanisms underlying risk of colorectal cancer from smoking and red/processed meat carcinogens by modeling exposure in normal colon organoids

SUPPLEMENTARY MATERIALS

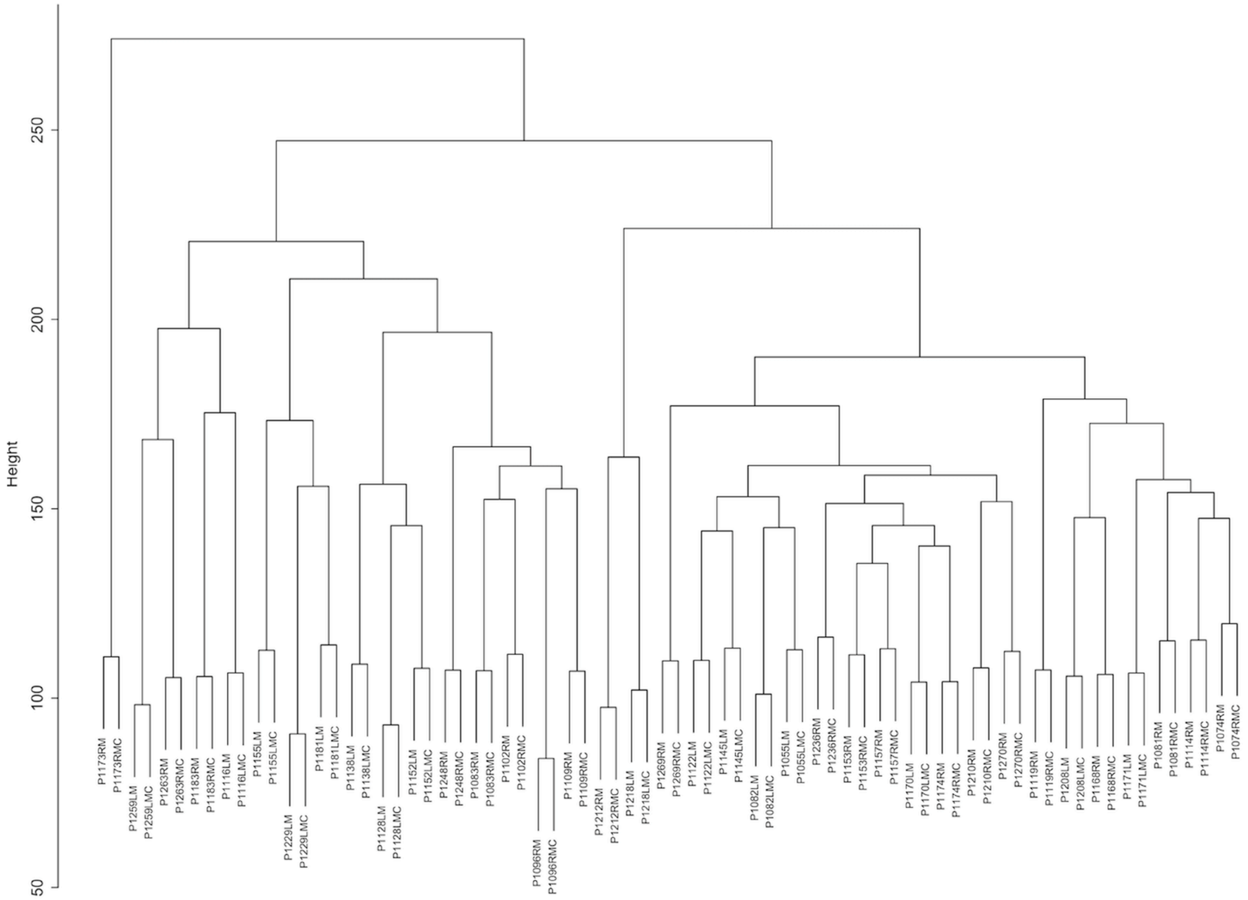

Supplementary Figure 1: Heirarchical clustering of colon organoids. Clustering revealed one outlier pair, which was subsequently removed from all downstream regression analysis.

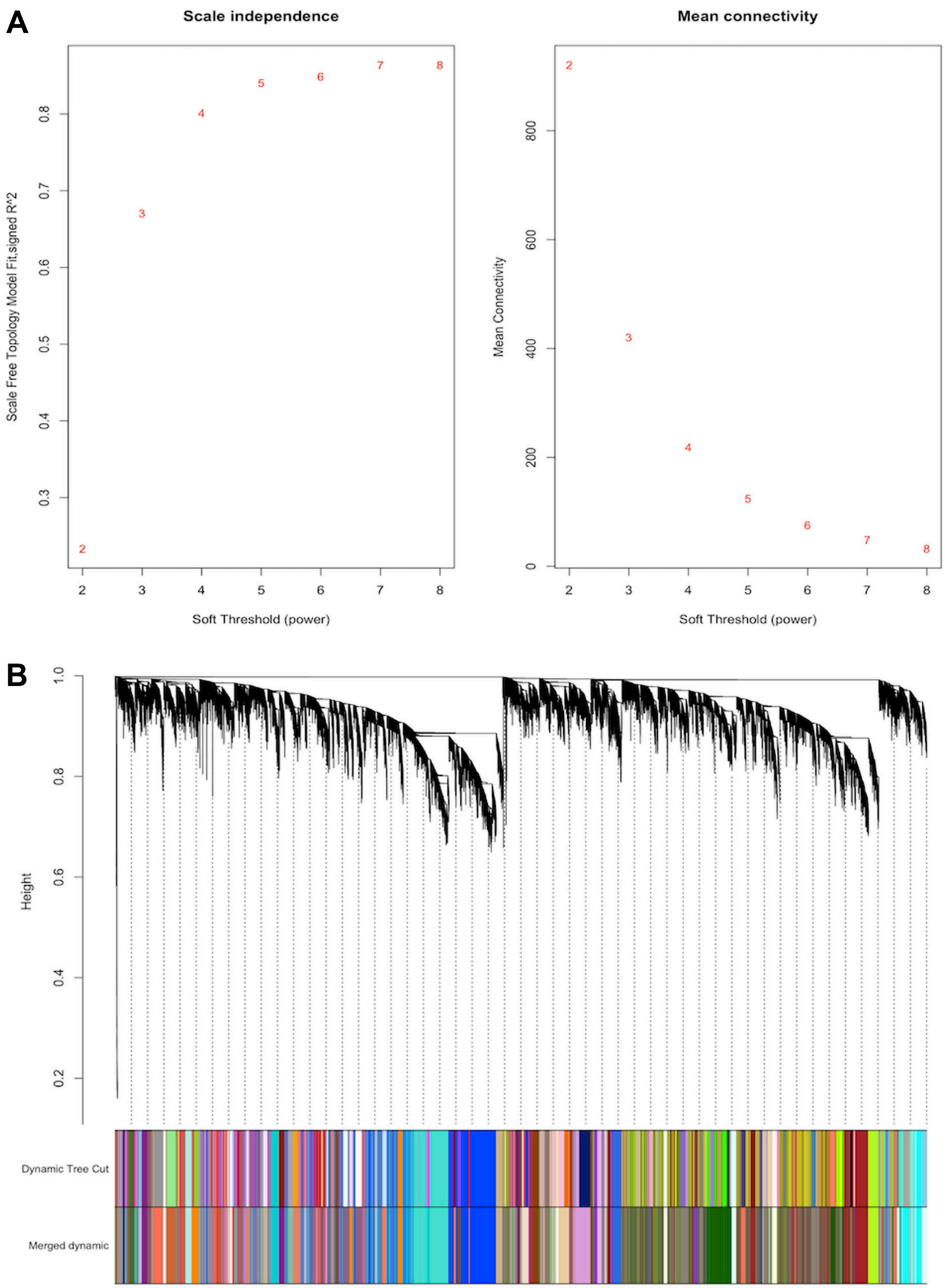

Supplementary Figure 2: WGCNA of carcinogen-treated colon organoids. (**A**) A soft power threshold of six was set to raise the network based on scale-free topology measures. (**B**) Clustering was performed to merge closely related modules prior to correlation with carcinogen treatment status.

Supplementary Table 1: Subject demographics of 36 colon organoid pairs considered for analysis

Individual	Sex	Age	Smoking Status	Biopsy Location
**1**	Female	22	Never	Left
**2**	Male	34	Former	Right
**3**	Male	66	Never	Right
**4**	Female	61	Current	Left
**5**	Male	59	Never	Right
**6**	Male	53	Never	Right
**7**	Female	71	Never	Right
**8**	Female	58	Never	Right
**9**	Male	64	Never	Right
**10**	Female	64	Never	Left
**11**	Male	54	Never	Right
**12**	Female	74	Never	Left
**13**	Male	56	Former	Left
**14**	Female	67	Former	Left
**15**	Male	73	Never	Left
**16**	Female	64	Never	Left
**17**	Male	61	Current	Right
**18**	Female	50	Former	Left
**19**	Male	63	Current	Right
**20**	Male	65	Never	Right
**21**	Female	62	Never	Left
**22**	Male	68	Current	Left
**23**	Female	70	Never	Right
**24**	Female	52	Current	Right
**25**	Female	50	Never	Left
**26**	Female	47	Never	Right
**27**	Male	59	Never	Right
**28**	Female	53	Never	Left
**29**	Male	32	Never	Right
**30**	Male	50	Never	Right
**31**	Male	51	Never	Left
**32**	Female	62	Never	Right
**33**	Female	63	Never	Right
**34**	Female	60	Never	Right
**35**	Female	50	Never	Left
**36**	Female	60	Former	Left
**37**[#]	Female	70	Never	Right

[#]Sample pair was excluded following results from initial regression analysis.

Supplementary Table 2: Summary of permutation analysis

N	Mean No. DEGs	Coefficient of Variation	No. DEGs versus Overall Cohort (%)
5	121 [24–355]	0.767	4.12
10	377 [192–621]	0.337	12.81
15	800 [480–1085]	0.194	27.17
20	1299 [1022–1746]	0.132	44.12
25	1856 [1628–2143]	0.080	63.05
30	2406 [2246–2686]	0.050	81.72

Squared brackets contain the minimum and maximum number of DEGs identified across the 20 permutations. Average number of DEGs were rounded to the nearest whole number. Given the number of permutations for each regression, limma to Dream for its considerably improved speed and reduced memory consumption. As such, the percentage of DEGs were calculated against a total that was also generated using limma (data not shown).

Supplementary Table 3: Summary of qPCR analysis

Gene	Assay	Estimate	Standard Error	*t*-value	*P*
DISP2	Hs00394338_m1	1.028	0.085	12.036	1.23E-03
BZW2	Hs00204063_m1	–0.486	0.058	–8.339	3.62E-03
UGT1A6	Hs01592477_m1	1.005	0.126	7.952	4.15E-03
ABCB1	Hs00184500_m1	0.62	0.126	4.922	0.016
MYEOV	Hs00993153_g1	–0.479	0.107	–4.468	0.021

Positive estimates indicate increased fold change in carcinogen treated colon organoids. For each gene Glucuronidase Beta (Hs00939627_m1) was used as an internal control.

Supplementary Table 4: Summary of carcinogen exposure-related DEGs overlapping genes mapping to CRC GWAS loci

Ensembl	HGNC	Mapped SNP	Chrom	Start (bp)	End (bp)	logFC	*t*	Pbonferroni	z.std
ENSG00000166908	PIP4K2C	rs4759277	12	57591174	57603418	0.343	11.221	3.83E-10	7.658
ENSG00000076685	NT5C2	rs4919687	10	103087185	103277605	0.224	9.338	7.34E-08	6.951
ENSG00000146192	FGD2	rs9470361	6	37005646	37029069	0.894	8.647	1.77E-07	6.826
ENSG00000137312	FLOT1	rs3131043	6	30727709	30742732	–0.310	–8.546	2.27E-07	–6.790
ENSG00000074527	NTN4	rs11108175	12	95657807	95791189	0.496	9.757	1.80E-06	6.485
ENSG00000124762	CDKN1A	rs9470361	6	36676460	36687337	0.357	8.397	2.34E-06	6.445
ENSG00000169174	PCSK9	rs12144319	1	55039447	55064852	–0.615	–8.780	2.42E-06	–6.440
ENSG00000169738	DCXR	rs373585858	17	82035136	82037709	0.360	7.864	2.58E-06	6.430
ENSG00000135111	TBX3	rs1427760	12	114670255	114684175	0.328	8.320	7.92E-06	6.258
ENSG00000204568	MRPS18B	rs3131043	6	30617840	30626395	–0.251	–7.582	9.97E-06	–6.222
ENSG00000127838	PNKD	rs3731861	2	218270392	218346793	–0.280	–7.676	1.33E-05	–6.176
ENSG00000088298	EDEM2	rs6058093	20	35115364	35147336	0.195	6.953	9.56E-05	5.857
ENSG00000108953	YWHAE	rs4968127	17	1344275	1400222	–0.151	–6.854	1.43E-04	–5.789
ENSG00000101190	TCFL5	rs1741640	20	62841005	62861822	0.272	6.808	1.51E-04	5.780
ENSG00000137491	SLCO2B1	rs7946853	11	75100563	75206549	0.298	7.663	3.48E-04	5.638
ENSG00000273340	MICE	rs1476570	6	29741731	29748969	–0.762	–6.590	8.34E-04	–5.486
ENSG00000167996	FTH1	rs174533	11	61959718	61967634	0.244	6.705	8.90E-04	5.474
ENSG00000087088	BAX	rs12979278	19	48954815	48961798	0.237	6.361	9.90E-04	5.455
ENSG00000188786	MTF1	rs4360494	1	37809574	37859592	0.171	6.353	1.47E-03	5.385
ENSG00000134243	SORT1	rs2938616	1	109309568	109397918	0.213	6.476	1.56E-03	5.374

ENSG00000152700	SAR1B	rs4976270	5	134601149	134649271	0.222	6.173	1.76E-03	5.352
ENSG00000075568	TMEM131	rs11692435	2	97756333	97995948	0.185	6.238	1.81E-03	5.347
ENSG00000125520	SLC2A4RG	rs2738783	20	63739776	63744050	0.320	6.138	1.93E-03	5.336
ENSG00000214922	HLA-F-AS1	rs1476570	6	29726601	29749049	–0.645	–6.634	1.96E-03	–5.332
ENSG00000167601	AXL	rs1963413	19	41219223	41261766	–0.667	–6.608	2.33E-03	–5.301
ENSG00000096070	BRPF3	rs9470361	6	36196744	36232790	0.144	5.973	3.52E-03	5.225
ENSG00000204386	NEU1	rs2516420	6	31857659	31862905	0.231	6.141	3.53E-03	5.225
ENSG00000143093	STRIP1	rs2938616	1	110031577	110074641	0.184	5.972	3.81E-03	5.211
ENSG00000124126	PREX1	rs6066825	20	48624252	48827999	–0.632	–6.073	4.57E-03	–5.177
ENSG00000173926	MARCHF3	rs12659017	5	126867714	127030558	0.241	6.081	4.72E-03	5.171
ENSG00000141580	WDR45B	rs373585858	17	82614562	82648553	–0.179	–6.110	5.20E-03	–5.153
ENSG00000101216	GMEB2	rs2738783	20	63587602	63627101	–0.203	–5.865	5.30E-03	–5.149
ENSG00000103591	AAGAB	rs12594720	15	67200667	67255195	0.174	6.037	6.19E-03	5.120
ENSG00000135108	FBXO21	rs55990915	12	117141991	117190471	0.169	5.842	7.19E-03	5.092
ENSG00000179912	R3HDM2	rs4759277	12	57253762	57431005	0.192	5.896	7.44E-03	5.085
ENSG00000101460	MAP1LC3A	rs6058093	20	34546854	34560345	0.220	5.800	7.99E-03	5.072
ENSG00000174358	SLC6A19	rs78368589	5	1201595	1225111	1.038	6.011	8.59E-03	5.058
ENSG00000183010	PYCR1	rs373585858	17	81932384	81942412	–0.261	–5.941	9.10E-03	–5.047
ENSG00000285761		rs1476570	6	29752573	29763295	–0.936	–5.839	0.012	–4.992
ENSG00000134824	FADS2	rs174533	11	61792980	61867354	–0.591	–6.142	0.012	–4.989
ENSG00000186115	CYP4F2	rs34797592	19	15878023	15898077	–0.523	–5.949	0.013	–4.975
ENSG00000133121	STARD13	rs377429877	13	33103137	33350630	–0.273	–5.735	0.015	–4.951
ENSG00000110925	CSRNP2	rs12372718	12	51061205	51083664	0.189	5.708	0.016	4.942
ENSG00000072958	AP1M1	rs34797592	19	16197854	16245906	–0.231	–5.648	0.017	–4.927
ENSG00000197142	ACSL5	rs12246635	10	112374018	112428380	0.174	5.617	0.017	4.923
ENSG00000166272	WBP1L	rs4919687	10	102743948	102834516	0.124	5.581	0.018	4.918
ENSG00000140577	CRTC3	rs7495132	15	90529923	90645345	0.179	5.536	0.018	4.917
ENSG00000161558	TMEM143	rs12979278	19	48332356	48364059	–0.243	–5.611	0.019	–4.900
ENSG00000105323	HNRNPUL1	rs1963413	19	41262496	41307787	–0.135	–5.579	0.020	–4.897
ENSG00000131941	RHPN2	rs28840750	19	32978592	33064888	–0.210	–5.718	0.022	–4.879
ENSG00000138175	ARL3	rs4919687	10	102673731	102714397	0.182	5.496	0.023	4.870
ENSG00000114573	ATP6V1A	rs72942485	3	113747033	113812056	0.183	5.514	0.027	4.834
ENSG00000168394	TAP1	rs2516420	6	32845209	32853816	0.205	6.013	0.031	4.805
ENSG00000182199	SHMT2	rs4759277	12	57229573	57234935	–0.206	–5.491	0.032	–4.799
ENSG00000163481	RNF25	rs3731861	2	218663892	218672002	0.273	5.406	0.036	4.779
ENSG00000183317	EPHA10	rs4360494	1	37713880	37765133	–0.242	–5.420	0.042	–4.746
ENSG00000119514	GALNT12	rs34405347	9	98807670	98850081	–0.156	–5.704	0.043	–4.742
ENSG00000227036	LINC00511	rs983318	17	72290091	72640472	0.189	5.391	0.045	4.733
ENSG00000116396	KCNC4	rs2938616	1	110210314	110283100	–0.273	–5.288	0.046	–4.728
ENSG00000162144	CYB561A3	rs174533	11	61348754	61362283	0.271	5.495	0.046	4.728
ENSG00000168658	VWA3B	rs11692435	2	98087116	98313299	–0.799	–5.254	0.046	–4.727

Chromosomal coordinates were generated for each gene of interest.

Supplementary Table 5: Parameters for single cell deconvolution of bulk RNA-seq

Stage	Settings	Choice
Signature Matrix Definition	Minimum Expression	0.2
	Sampling	1
	Barcode gene range	300-500
	Q-value	0.05
Quantification	Batch correction	S-mode
	Absolute Mode	True
	Permutations	1000

Default arguments for parameters are not listed.

Supplementary File 1: Summary mapping statistics for each sample used within the colon organoid study. See Supplementary File 1

Supplementary File 2: The overlap between carcinogen DEGs and those observed in the literature (as collated by the Comparative Toxicogenomics Database) as well as through novel analysis of smoking and red meat are provided. See Supplementary File 2

Supplementary File 3: File contains the covariates used for BarcUVa-Seq regression analyses employed within the manuscript. See Supplementary File 3

Supplementary File 4: File contains enriched GO terms identified through pathway analysis of each significant WGCNA module. See Supplementary File 4
